# Transcriptomic Characterization of *Bradyrhizobium diazoefficiens* Bacteroids Reveals a Post-Symbiotic, Hemibiotrophic-Like Lifestyle of the Bacteria within Senescing Soybean Nodules

**DOI:** 10.3390/ijms19123918

**Published:** 2018-12-07

**Authors:** Sooyoung Franck, Kent N. Strodtman, Jing Qiu, David W. Emerich

**Affiliations:** 1 Division of Biochemistry, University of Missouri, Columbia, MO 65211, USA; sooyoungfranck@live.com (S.F.); knstrodtman@ccis.edu (K.N.S.); 2 Applied Economics and Statistics, University of Delaware, Newark, DE 19716, USA; qiujing@udel.edu

**Keywords:** *bradyrhizobium diazoefficiens*, soybean, Glycine max, nitrogen fixation, senescence, transcriptomics, hemibiotroph

## Abstract

The transcriptional activity of *Bradyrhizobium diazoefficens* isolated from soybean nodules was monitored over the period from symbiosis to late plant nodule senescence. The bacteria retained a near constant level of RNA throughout this period, and the variation in genes demonstrating increased, decreased, and/or patterned transcriptional activity indicates that the bacteria are responding to the changing environment within the nodule as the plant cells progress from an organized cellular structure to an unorganized state of internal decay. The transcriptional variation and persistence of the bacteria suggest that the bacteria are adapting to their environment and acting similar to hemibiotrophs, which survive both as saprophytes on live plant tissues and then as necrophytes on decaying plant tissues. The host plant restrictions of symbiosis make *B*. *diazoefficiens* a highly specialized, restricted hemibiotroph.

## 1. Introduction

Soybean nodules are symbiotic organs that are formed on roots by the complex interaction between soybean plants and *rhizobia*, nitrogen-fixing bacteria, under nitrogen-limiting conditions. There are two types of nodules found on leguminous plants: determinate and indeterminate [1,2,3,4,5]. Determinate nodules, such as those formed by *Bradyrhizobium diazoefficiens* and soybean, form a determinate, developmentally synchronized, nitrogen-fixing symbiosis, that is, they predominately contain one specific developmental form of the bacteria that is dictated by the nodule’s age [6,7]. Thus, all of the nodules within the crown of the root are of the same developmental age. Indeterminate or longitudinal nodules, such as those found on alfalfa formed by infection with *Sinorhizobium meliloti*, form structures that vary in age from the growing point that contains the youngest material to the base of the nodule that contains the oldest. Indeterminate nodules contain a mixture of all bacterial and bacteroid forms in all stages of development. Differentiated *Rhizobia* in a root nodule are referred as bacteroids, and they exchange fixed nitrogen to the plant in return for carbon sources from the host plants to provide the energy for the nitrogen fixation process. Extensive studies have been performed regarding the establishment of a nodule and the nitrogen fixation process between soybean plants and *B. diazoefficiens* [1,2,3,4,5].

Soybean nodule development resulting in mature nitrogen-fixing nodules is a programmed series of molecular events that are performed synchronously by both symbionts [1,2,3,4,5]. To maintain synchrony throughout symbiosis, the two symbionts must communicate continuously and function harmoniously at multiple levels. Senescence is a programmed physiological process following symbiosis, which breaks the inter-kingdom synchrony of the symbiosis. The bacteroids of the two types of nodules have different fates during senescence [8,9,10,11,12].

Indeterminate nodules, such as those of *Medicago truncatula* bacteroids, were observed to degrade at the first sign of plant nodule senescence [11,12]. In determinate soybean nodules, visual degradation of bacteroids was not observed, bacteroid protein content did not change over senescence, but remained constant, and degradative changes in bacteroids were not detected [10]. Strodtman et al. [13] found that soybean nodule bacteroids actively synthesize appendages up to 95 days after planting. Soybean nodule bacteroids are known to be viable at the end of the growing season, and capable of redifferentiation into free-living bacteria [1,5,9,11,14,15,16].

Several transcriptomic analyses of free-living *B. diazoefficiens* cells [17,18,19] and bacteroids [17,20,21] have already been performed using custom oligonucleotide microarrays to identify global gene expression patterns. Van de Velde et al. [12] have reported a microarray analysis of nodule senescence of the legume *Medicago truncatula*, but not of the bacterial symbiont, which has been demonstrated to degrade during senescence. Previous reports on soybean nodule bacteroids focused on a single time point during optimal symbiotic functioning [17,20,21]. This report presents a multiple time-point microarray study in *B. diazoefficiens* bacteroids isolated from soybean nodules from early symbiosis through late senescence to determine the genes that are differentially expressed by *B. diazoefficiens* bacteroids during senescence.

## 2. Results

### 2.1. Biological Parameters of Symbiosis and Senescence

**Figure 1:**
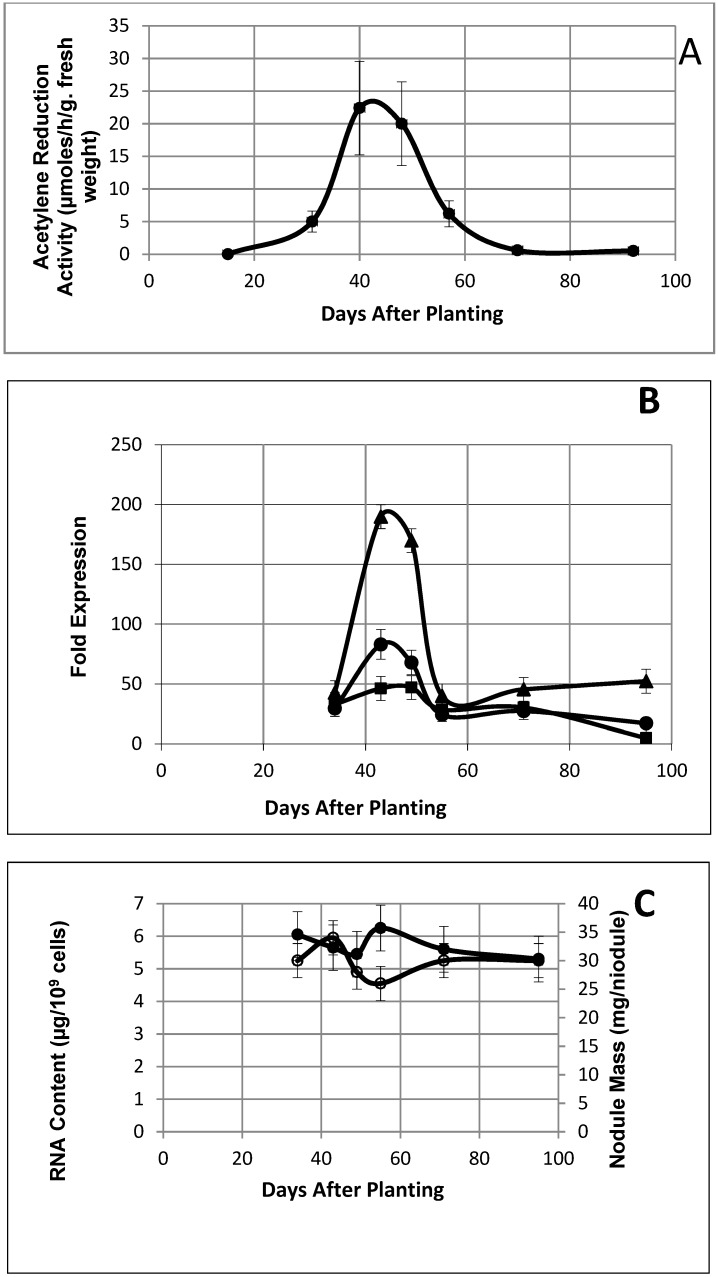
(**A**) Acetylene reduction activity of soybean nodules as a function of days after planting. Values are the means of three replicates. (**B**) Nitrogenase component gene expression from microarray data as a function of days after planting. Symbols represent the molybdenum–iron protein, alpha subunit (●); molybdenum–iron protein, beta subunit (■); iron protein (▲). Each value is the average of three samples ± S.D. (**C**) Total RNA isolated from soybean nodule bacteroids and soybean nodule mass as a function of days after planting. Values are means of at least three replicates. Solid symbols are RNA content; open symbols are nodule mass.

The nitrogen fixation activity of soybean nodules as measured by the acetylene reduction technique showed the typical pattern of a determinate symbiosis providing a reference frame by which to evaluate gene expression (Figure 1A). The nitrogen fixation activity peaked at around 40 days after planting and was negligible by day 70 (Figure 1A).

The expression of the genes for the nitrogenase component proteins showed the same profile as the nitrogen fixation activity as measured by the acetylene reduction activity of whole nodules (Figure 1B).

**Figure 2:**
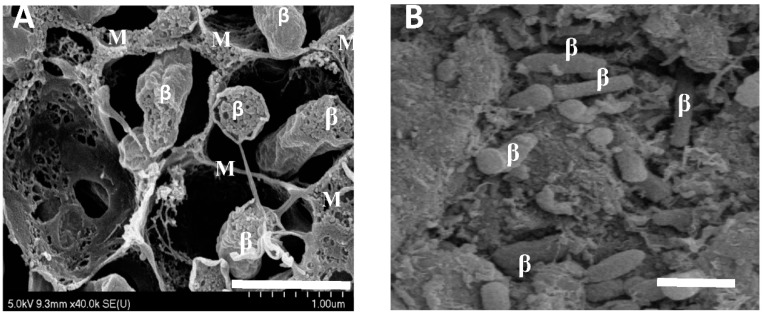
Scanning electron micrographs of soybean nodules 42 and 95 days after planting. (**A**) A soybean nodule at 42 days after planting. Bar represents 1 μm. (**B**) A soybean nodule at 95 days after planting. Bar represents 1 μm. Bacteroids are indicated as “β”; membranes and cellular material are indicated by “M”.

Bacteroids isolated from soybean nodules demonstrated a relatively constant RNA content over the 61 day period from 34 to 95 days after planting (Figure 1C), thus eliminating the need to normalize the gene expression data with regard to the RNA content. The RNA content of *B*. *diazoefficiens* cultured on an HM-Arabinose medium was 19.3 ± 3.7 µg/10^9^ cells. Although the nodule mass remained fairly constant to 95 days after planting (Figure 1C), the nodule’s exterior became noticeably lignified by day 55 as verified by phloroglucinol staining, and there was extensive disintegration of the interior plant cellular structure (Figure 2).

### 2.2. Global Gene Expression

**Figure 3:**
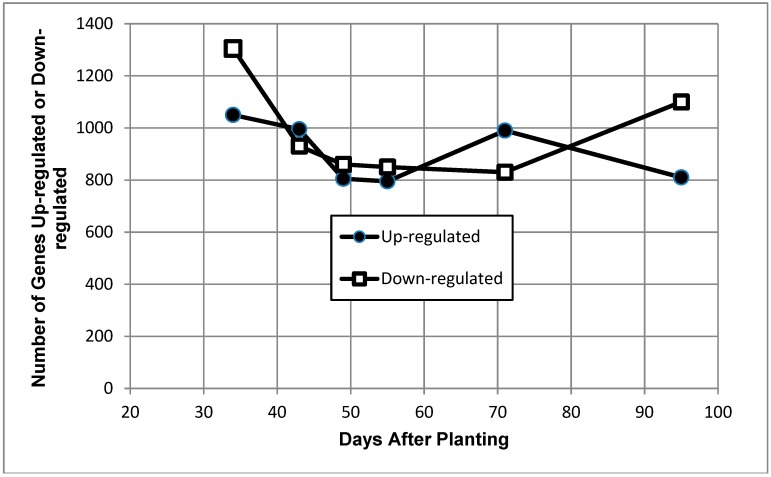
Number of up- and down-regulated genes of *Bradyrhizobium diazoefficiens* bacteroids as a function of days after planting. The transcripts included at each time point were determined independently of their values at other time points; thus, transcripts are typically included in multiple time points and may occur in either category. Solid symbols are up-regulated genes, and open symbols are down-regulated genes.

Of the 8480 genes analyzed, >800 were up- or down-regulated at each time point, with the up-regulated gene numbers showing less variation than the down-regulated genes, which demonstrated notable increases in numbers at the earliest and the latest time points relative to the other time points (Figure 3).

More than 60% of all the up-regulated genes were in the Hypothetical or Other Functions categories (Table 1). There was considerable variation in the composition of each time point of the up- and down-regulated gene pools, resulting in a variety of patterns. The maximum observed value of expression was +196-fold (blr6123, hypothetical) and the minimum value of expression observed was—152-fold (bll5085, hypothetical). The large volume and complexity of the data compelled that the analysis be restricted to a few highly repetitive patterns containing individual transcripts with uniform, overlapping expression values.

### 2.3. Expression Patterns: Constitutive

**Figure 4:**
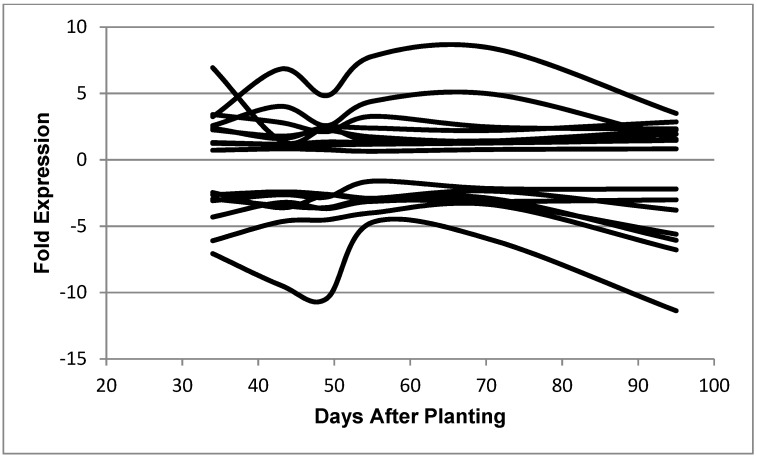
Expression of *B*. *diazoefficiens* bacteroid genes related to transcription as a function of days after planting. Fold expression of the transcription-related genes at 95 days after planting: RNA polymerase sigma-54 subunit (3.50), putative RNA polymerase sigma factor protein (2.86), RNA polymerase sigma-54 subunit (2.33), sigma32-like factor (2.25), RNA polymerase sigma-E factor (1.93), sigma-54 modulation protein (1.55), ECF sigma factor (1.49), ECF family sigma factor (1.47), RNA polymerase (0.82), cold-shock dead-box protein A (−2.20), DNA-directed RNA polymerase beta chain (−3.01), DNA-directed RNA polymerase beta chain (−3.79), RNA polymerase omega subunit (−5.60), transcription anti-termination protein (−6.06), DNA-directed RNA polymerase alpha subunit (−6.78), and ATP-dependent RNA helicase (−11.38).

**Figure 5:**
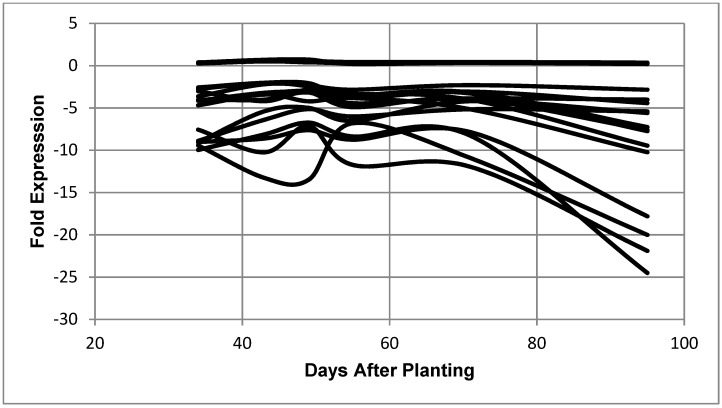
Expression of *B*. *diazoefficiens* bacteroid 30S ribosomal proteins as a function of days after planting. Fold expression value of 30S ribosomal proteins at 95 days after planting: S16 (0.36), S2 (0.29), S20 (0.27), S4 (0.20), S13 (0.19), S12 (−2.85), S19 (4.02), S6 (−4.43), S8 (−5.41), S14 (−5.60), S18 (−7.27), S7 (−7.61), S11 (−7.74), S1 (−9.46), S15 (−10.24), S3 (−17.81), S21 (−20.02), S10 (−21.90), and S17 (−24.51).

**Figure 6:**
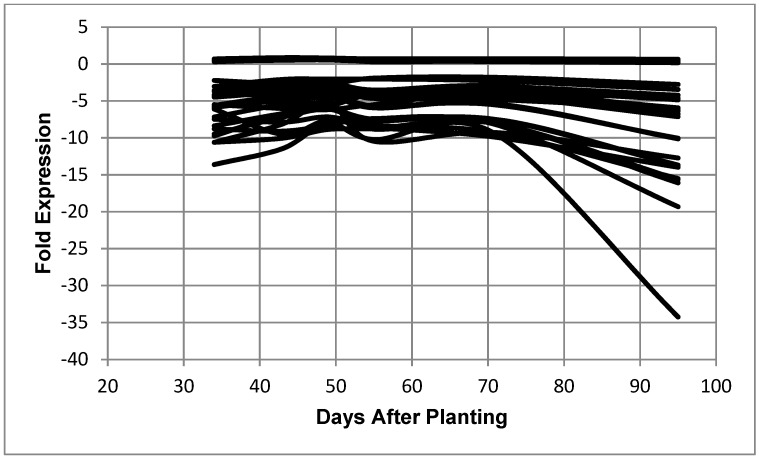
Expression of *B*. *diazoefficiens* bacteroid 50S ribosomal proteins as a function of days after planting. Fold expression value of 50S ribosomal proteins at 95 days after planting: L18 (0.65), L27 (0.40), L7/12 (0.38), L31 (0.33), L19 (0.20), L28 (0.17), L10 (−2.76), L21 (−3.44), L25 (−4.22), L13 (−4.65), L11 (−4.82), L2 (−4.86), L23 (−5.93), L9 (−6.07), L17 (−6.21), L15 (−6.38), L22 (−6.76), L20 (−7.13), L1 (−10.04), L35 (−10.13), L6 (−12.72), L24 (−13.68), L14 (−13.71), L3 (−13.98), L29 (−15.50), L5 (−15.63), L4 (−16.09), L16 (−19.32), and L30 (−34.25).

A significant proportion of the transcripts demonstrated a constitutive expression within the range of <±2 relative to the exponentially growing cultured cells over the entire time course of 61 days (Table 1). This narrowly defined category of constitutively expressed genes was ~11% of the total genome. More than 50% of the constitutive genes were categorized as Hypothetical or Other Functions. Interestingly, a number of the genes involved in transcription (Figure 4) and translation (Figure 5 and Figure 6) demonstrated a constitutive pattern. The gene expression profiles of the transcription and translation category also represent examples of constitutive gene expression outside of the <±2 boundaries. The most notable differences among the three groups listed in Table 1 were that (i) a larger percentage of amino acid metabolism and cell envelope genes was down-regulated; (ii) a smaller percentage of central intermediary metabolism was down-regulated, and (iii) a smaller percentage of cellular processes; fatty acid, phospholipid, and sterol metabolism; purines and pyrimidines; nucleosides and nucleotides; and translation genes was up-regulated.

### 2.4. Expression Patterns: Nitrogen-Fixation-Related

**Figure 7:**
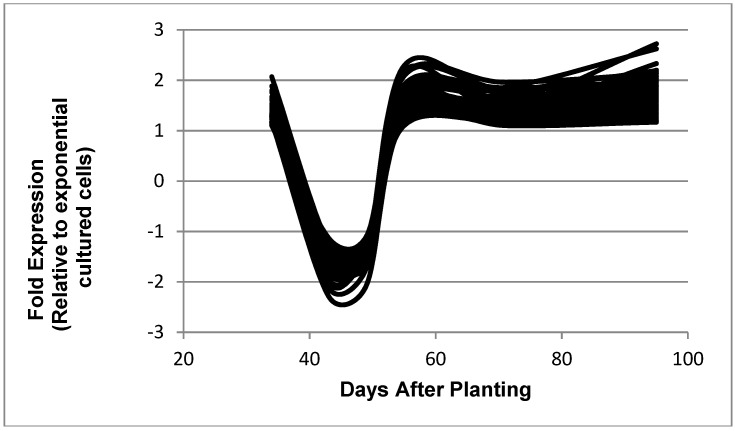
Expressed genes representing the reciprocal symbiosis pattern. The 68 genes shown in the figure are: bll0116, bll0195, bll0776, bll0979, bll1109, bll1422, bll2116, bll2410, bll2494, bll2675, bll3374, bll3444, bll3470, bll3505, bll3783, bll4141, bll4201, bll4403, bll4596, bll4793, bll5240, bll5296, bll5959, bll6302, bll6471, bll6498, bll6891, bll7298, bll7311, bll7402, bll7610, bll7835, bll7964, blr0310, blr0925, blr1457, blr2533, blr2715, blr2809, blr3324, blr3400, blr3517, blr4155, blr4311, blr4566, blr4772, blr5162, blr5169, blr5229, blr6118, blr6544, blr6569, blr6661, blr6771, blr7060, blr7216, blr7281, blr7496, blr7595, blr7717, blr7815, blr8000, blr8060, blr8194, bsl3895, bsl7758, bsr1887, and bsr3197.

**Figure 8:**
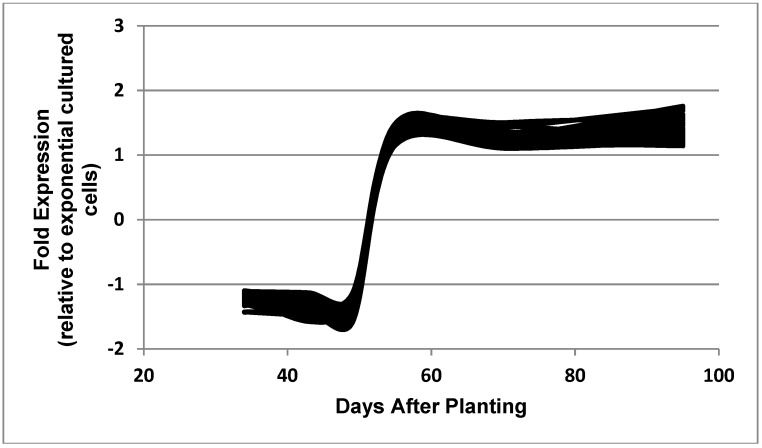
Expressed genes representing the post-symbiotic category. The 33 genes shown in the figure are: bll3172, bll4771, bll6387, bll6597, bll8078, blr0490, blr0998, blr2666, blr2834, blr2882, blr2923, blr3110, blr3499, blr3603, blr3634, blr3898, blr4361, blr5112, blr7482, blr7579, bsr1885, bll0052, bll0242, bll0244, bll0493, bll1533, bll2075, bll2188, bll4292, bll6051, blr4337, blr7208, and blr7482.

A number of patterns were identified to be relevant to the pattern of transcripts for the nitrogenase genes: (i) a symbiosis pattern that was the same as that represented by the nitrogenase component proteins (Figure 1B, Table 2); (ii) a reciprocal-symbiosis pattern in which the expression values at days 43 and 49 decrease, whereas the expression values at the other time points remain similar (Figure 7, Table 3); and (iii) a post-symbiotic pattern in which the expression levels remain constant at 34, 43, and 49 days and then increase significantly and remain similar at 55, 71, and 95 days (Figure 8, Table 4). These three patterns relate to nitrogen fixation, which is the primary physiological function of soybean nodules. Other patterns, such as those demonstrating significant increases at 71 days or 95 days, are largely hypothetical or unknown proteins. Among the functionally annotated genes expressed at 71 and 95 DAP are several ABC transport ATP-binding proteins, including bll3190, blr3340, and blr3544 and the α-ketoglutarate permease bll2904, and several proteins of sulfur metabolism, including the glutathione S-transferase-like protein bll4398, the cystathionine beta lyase bll4445, and the cysteine synthase bll4453. The genes that are more actively transcribed at 95 DAP include the regulatory proteins blr2424, blr5109, and blr0155, the ABC permease proteins blr5304 and blr7869, the potassium uptake protein blr3802, the small heat shock protein blr5220, and the metabolic proteins, including the phospholipid N-methytransferase blr0681, the acetyl CoA synthase blr3924, the indolepyruvate ferredoxin oxidoreductase, alpha subunit, bll3411, the ribulose 1,5-bisphosphate carboxylase/oxygenase small subunit blr2586, the gluconolactonase precursor bll2956, the 3-oxoadipate CoA-transferase subunit A bll3462, and the glutathione reductase blr3757. These genes add to the composite of the genes represented in the other patterns, demonstrating a metabolically viable micro-organism.

The symbiotic pattern included the nitrogenase component proteins (Figure 1B) as well as many other previously noted symbiotic-related proteins (Table 2) [17,20]. The genes expressed that demonstrate the symbiotic pattern include proteins found in the cytoplasm, cytoplasmic membrane, outer membrane, and periplasm (Table 2). The difference between the minimum and maximum expression values of each profile for the symbiotic pattern averaged 9.30-fold. As with the symbiotic pattern, genes in the reciprocal symbiosis pattern include proteins found both intra- and extracellularly (Table 3). The difference between the minimum and maximum expression of each profile for the reciprocal symbiotic pattern values averaged 2.88-fold. The post-symbiotic pattern shown in Figure 8 shows a rapid transition between 49 and 55 that correlates with the decline of nitrogenase activity. The post-symbiotic pattern also includes proteins found both intra- and extracellularly (Table 4). The difference between the minimum and maximum expression values of each profile for the post-symbiotic pattern averaged 2.62-fold.

### 2.5. Molecular Signatures

Molecular signatures, including the frequency of rare codons, %GC, %AG, and % of each of the four nucleotides, were examined to identify a correlation with each expression pattern and functional group. When all of the rare codons were compared as a group, the symbiotic pattern was statistically different from each of the other three patterns, and the constitutive pattern was statistically different from the other three respective patterns; however, the post-symbiotic and the reciprocal symbiotic patterns were not statistically different from each other (Table 5). The symbiotic, post-symbiotic, and constitutive expression patterns were all statistically different from the three functional groups (transcription, 30S and 50S ribosomal genes); however, the reciprocal symbiotic pattern was not. The ribosomal genes contained relatively few rare codons compared to the expression patterns.

The constitutive group showed statistically greater %GC than the genes expressed during symbiosis and post-symbiosis (Table 6). The symbiotically expressed genes had statistically significantly greater %AG contents than the other expression patterns and functional groups. The reciprocal and post-symbiotic patterns were not statistically different from each other in any category. There were multiple significant differences in the %A and %T contents of transcripts among the expression patterns and functional groups, and only a few such differences with regard to the %G and %C.

## 3. Discussion

The gene expression of the symbiotic *Bradyrhizobium diazoefficiens* bacteria during symbiosis has been only reported at single time points [17,20,21], but not over an extensive time interval from symbiosis through senescence. The relatively constant level of *B*. *dizoefficiens* RNA obtained over the time period of the measurements allows for a more direct comparison of changes that occurred among the time points. Such measurements over extended time intervals can yield more information about symbiosis and senescence. For example, our previous study identified 150 reiterated genes among three independent research teams based on single time points [20]. Of these 150 genes, 130 were found here to demonstrate greater than 2-fold expression at all six time points; however, only 45 were congruent with the symbiosis pattern as defined here (Figure 1B). Thus, multiple time points would help to differentiate genes that correlate with a particular long-term physiological pattern of nodule development and senescence rather than short-term environment events.

Of the 8480 open reading frames represented on the microarray [17], approximately 20–30% were defined as either up-regulated or down-regulated at each of the six time points (Table 1, Figure 3). A large proportion of the transcripts were unchanged or constitutively expressed from 34 to 95 days after planting. Pfeiffer et al. [10] reported that the total soluble bacteroid protein per gram of nodule remained constant through to harvest maturity of soybeans (~120–150 days after planting). These constitutively expressed genes include those that are necessary for cellular maintenance, such as those for transcription (Figure 4) and translation (Figure 5 and Figure 6). The ribosomal genes showed constitutive expression to 95 days after planting, many not significantly different from the controls, which were exponentially grown cultured cells. These results indicate that the bacteria are transcriptionally and translationally active to 95 days after planting and suggest that the bacteria adjust to conditions in the nodule over time after the completion of nitrogen fixation.

Berthoumieux et al. [23] determined that the physiological state of the cell has a profound influence on gene expression. The unique, robust physiology imposed by symbiotic nitrogen fixation was evident in the transcriptome [17,20,21]. Genes expressed during the symbiotic pattern demonstrated a greater fold expression (9.3 versus 2.88 and 2.62) than the other patterns. Despite the impact of symbiotic nitrogen fixation on the transcriptome, after the nitrogen fixation activity of the nodule declined, the amount of total bacteroid RNA remained relatively the same as during active nitrogen fixation. Other genes became expressed and persisted to 95 days after planting, presumably to cope with the environment of the senescent nodule. The post-nitrogen fixation period displayed two well-defined patterns: the post-symbiotic pattern and the reciprocal symbiosis pattern. These two patterns were similar to each other (Figure 7 and Figure 8) with the exception of the level of gene expression of the first time point at 34 days. Thus, the only difference between the two patterns may be at the early stages of symbiosis. However, the highly repetitive nature and number of the expression patterns in these two groups demonstrates the robustness of the post-symbiotic transformation. During the period of nodule development and senescence, the plant portion of the nodule underwent a drastic change from a functional, structurally intact organ to a lignified shell devoid of an internal structure with only the former symbiotic bacteria intact (Figure 2). The entrapped bacteria retained their transcriptional activity. They also synthesize appendages [13], and the protein [10] and RNA levels (Figure 1C) remain constant. Strodtman et al. have shown that post-symbiotic bacteria remain metabolically active up to 119 days after planting [24]. The retention of transcriptional, translational [24], and metabolic [24] activities of the bacteria as the plant cells dies is behavior reminiscent of a hemibiotroph [25,26]. A hemibiotroph is an organism that is saprophytic or parasitic in living tissue while the plant is alive and upon plant death consumes the dead tissue [25,26]. Although *B*. *diazoefficiens* is a symbiont and not a parasite on living plant tissue, it survives on metabolites that the plant provides. The bacteria after symbiosis continue to function in decaying plant nodules by producing transcripts for genes encoding proteins found both intracellularly and extracellularly. *Bradyrhizobium diazoefficiens* should be considered a highly specialized hemibiotroph restricted to a single plant species and a single, specialized plant organ. The restrictions of its specific interactions are dictated by the restrictions placed upon it by the symbiosis.

An analysis of the transcripts in the three distinct categories, namely symbiosis, the hemibiotroph-like patterns consisting of the post-symbiotic pattern and the reciprocal symbiotic pattern, and the constitutive pattern, was performed to determine whether there were unique molecular signatures, beyond the expression patterns, that were analogous to the unique %GC composition of the symbiotic island [22]. The frequency of rare codons was greatest in the symbiotic pattern and lowest in the constitutive, transcription, 30S ribosome, and 50S ribosome genes. The hemibiotroph-like patterns were statistically different from the symbiotic and constitutive patterns; however, the post-symbiotic and reciprocal symbiotic patterns were not statistically different from each other. Quaz et al. [27] reported that codon bias is a means to modify or adjust gene expression. The differences may also reflect the acquisition of the symbiotic island from an ancient donor organism and that the constitutive, transcription, and translation genes are ancestral to *B*. *diazoefficiens*. The genes representing the hemibiotrophic-like patterns do not show clustering into a unique island like the symbiosis island, and, thus, may be ancestral, native traits. As previously noted by Frank et al. [17], the %GC content of genes is not sufficient to identify symbiotically relevant genes (Table 6). The %AG showed a statistical difference between the symbiotic expression pattern and the other three patterns. The hemibiotrophic-like patterns and the constitutive patterns were not statistically different from one another. Zuckerkandl [28] has reported that increased purine content of transcripts reduced secondary structural interactions, which may reflect on the lifetime and stability of the messages. Differences in the content of individual nucleotides in each category of gene expression were most evident for %A and %T for the symbiotic and constitutive categories. The post-symbiotic and reciprocal symbiotic categories were similar to each other for all nucleotides (Table 6). Wang and Hickey [29] reported that the 16S rRNAs favored purines, particularly adenine. This preference for adenine is particularly apparent for the 30S and 50S ribosomal genes (Table 6). Thus, for *B*. *diazoefficiens*, the preference for adenine extends beyond the 16S rRNA.

*B diazoefficiens* bacteroids underwent a global shift in gene expression patterns during the transition from symbiosis to nodule senescence (Figure 1B, Figure 7 and Figure 8), although their total RNA quantity and quality remained relatively unchanged (Figure 1C). The post-symbiosis and reciprocal symbiosis expression patterns plus the persistence of the transcription- and translation-related genes indicate that the bacteria remain metabolically viable within the decaying soybean nodule, suggesting a hemibiotrophic-like lifestyle. The large number of Hypothetical and Other Function genes expressed during senescence and the lack of unique molecular markers limit the characterization and interpretation of the lifestyle of *B*. *diazoefficiens* during soybean nodule senescence.

## 4. Materials and Methods

### 4.1. Fabrication of the Spotted Oligonucleotide Microarray for *B. diazoefficiens*

The GeneMark [30] and Glimmer2 [31] gene models were used to generate a list of unique, non-overlapping open reading frames (ORFs). Sequences of 8317 ORFs were predicted from the machine-annotated *B. diazoefficiens* strain USDA 110 genome [32]. An additional 226 ORFs of the genome were reannotated [17]. However, 63 ORFs derived from the original annotation were excluded due to the lack of suitable unique probes; thus, a total of 8480 ORFs was represented on the microarray slide. Each slide had the 8480 ORFs and 36 control genes printed in duplicate. For each predicted ORF, 70-mer oligonucleotides were designed and synthesized (Qiagen Operon, Valencia, CA, USA). The oligonucleotides were printed onto Corning^®^ epoxysilane-coated slides (Corning Inc., Lowell, MA, USA) at the Genome Sequencing Center at Washington University School of Medicine.

### 4.2. Plant Growth, Acetylene Reduction, Nodule Lignification, and Bacteroid Isolation

Soybean plants (Williams 82 inoculated with *B*. *diazoefficiens* 110) were grown in the field at the Bradford Research and Extension Center, Columbia, Missouri, and all the samples were harvested at mid-morning. Samples were collected at 34, 43, 49, 55, 71, and 95 days after planting. Each sampling included three biological replicates (~100 g each). The roots were immediately placed in ice water at the time of harvest, and the nodules were isolated in the cold room at 4 °C on the same day. The isolated nodules were kept frozen at −80 °C until bacteroid isolation. The color of the nodules started to change from light brown to light green at 55 days after planting, and the texture of the nodules became noticeably fragile on day 95 after planting. Nodulated root segments were removed from the soil, quickly trimmed to remove lateral roots leaving the nodules located at the crown (~1 inch below surface), placed in 20 mL serum bottles, capped, and 10% *v*/*v* acetylene was added to start the assay [32]. One milliliter gas samples were removed at 5-min intervals and stored until analyzed upon return to the laboratory. Ethylene formation was determined by gas chromatography [32]. The procedure of Mitra and Loque [33] was used to provide a qualitative analysis of nodule surface lignification. The procedure used for the isolation of bacteroids was essentially as described [34] except that the gradient consisted of 42% and 57% (*w*/*w*) sucrose in MEP buffer (5 mM MgCl_2_, 1 mM EDTA, 50 mM potassium phosphate buffer, pH 7.0).

### 4.3. Bacteria Growth and Media

*B. diazoefficiens* strains USDA110 were grown aerobically at 30 °C in HM medium [35] supplemented with 0.3% arabinose and 0.025% yeast extract (Difco), and used as controls in all of our reference-designed microarray experiments. Cells were grown to mid log phase (OD_630_~0.5), immediately cooled down in ice for 10 min in the cold room at 4 °C, collected by centrifugation (8000× *g*, 10 min), and washed with 0.2 M NaCl followed by a wash in distilled water. Three separate cultures and two technical replicates for each culture were analyzed.

### 4.4. RNA Isolation

The same procedures of RNA isolation were used both for the bacteria and bacteroids. All procedures were performed in a 4 °C cold room. Cells (~10 g each biological sample, two technical replications) were immediately transferred to centrifuge tubes containing cold stop solution (5% phenol (pH 4.3), 95% ethanol) and centrifuged for 10 min at 4 °C, 9700× *g*. The cell pellets were immediately frozen in liquid nitrogen and stored at −80 °C until RNA isolation. RNA was isolated using a hot phenol method as described previously [36] and treated with DNase I (10 Units, Promega, Madison, WI, USA) and RNase inhibitor (40 Units Promega) for 30 min at 37 °C followed by phenol-chloroform extraction and column purification using an RNeasy Mini Kit (Qiagene, Germantown, MD, USA). The integrity of all RNA samples was confirmed on 0.8% agarose gels.

### 4.5. cDNA Synthesis and Hybridization

Initial experiments to test the utility of the microarrays were performed at the Genome Sequencing Center in Washington University School of Medicine (http://genome.wustl.edu/services/microarray.cgi). cDNA was synthesized and labeled as described previously [37]. Each biological replicated sample was split into two technical replications. Twenty micrograms of total RNA was used for cDNA synthesis with superscript III reverse transcriptase and random hexamers (Invitrogen Corp., Waltham, MA, USA), an aminoacyl-dNTP mix, 10 mM DTT, and incubation overnight at 42 °C. To remove the remains of RNA, the sample was treated with RNase H (Fermentas, Waltham, MA, USA). The cDNA was further purified using a Microcon YM-30 column (Millipore-Sigma, Burlington, MA, USA ). The concentration was determined using a Nanodrop spectrophotometer (NanoDrop Technologies, Wilmington, DE, USA). cDNA (4.5 μg) was used for labeling with either cy3 or cy5 (Amersham, Buckinghamshire, UK), and unincorporated dyes were removed using a Qiaquick PCR purification Kit (Qiagen). Both cy3- and cy5-labeled cDNA samples were mixed, dried to completion, and resuspended in 70 µL of preheated hybridization buffer (nuclease free water:formamide:20X SSC:1% SDS = 4:2.5:2.5:1) at 42 °C. To prevent non-specific binding of the samples to the array, 0.7 µL of salmon sperm DNA (10 mg/mL) was mixed in the samples. The mixture was hybridized at 42 °C for 16–18 h. The arrays were washed once with 1X SSC, 0.2% SDS at 42 °C for 6 min, once with 0.1 X SSC, 0.2% SDS at room temperature for 6 min, and twice with 0.1X SSC at room temperature for 3 min. The arrays were scanned using an Axon GenePix 4000B scanner (Molecular Devices Corp., Sunnyvale, CA, USA).

### 4.6. Data Analysis

The array was scanned using the GenePix^®^ Pro 6.0 software (Molecular Devices Corp., Sunnyvale, CA, USA), and saved as TIFF image files containing fluorescence signal intensities for each spot. The normalization method for spot and slide abnormalities was spatial LOWESS. A mixed-effect microarray analysis of variance (MAANOVA) [38,39] was used for the subsequent data normalization. The resulting variety-by-gene interaction values were combined with the residual noise observed from each spot to obtain the filtered and adjusted expression values [39]. Both LOWESS and MAANOVA are part of the R/maanova microarray statistical analysis package (http://www.jax.org/staff/churchill/labsite/). The expression values from two technical replicates in each array were averaged, and then subsequently subjected to a significance analysis of microarray (SAM) data using the SAM package software [40]. Significant genes were selected based on the genes with a false discovery rate lower than 5% (*q* = 0.05) at various threshold of fold change cut-offs. The data have been deposited in the Gene Expression Omnibus GSE60465.

### 4.7. Quantitative Reverse Transcription-PCR (qRT-PCR) Analysis

In order to validate the microarray data, the mRNA expression of 10 genes was measured by qRT-PCR using the same RNA samples as used in the microarray experiments [17]. Prior to the reverse transcriptase treatment, a total 3 μg of RNA was treated with Turbo DNase (Ambion, Foster City, CA, USA for 1 h at 37 °C, and reverse-transcribed into cDNA using a 10 mM dNTP mix and superscript III reverse transcriptase for 1 h at 42 °C. The quantitative PCR-amplification using SYBR^®^ Green Master Mix (Applied Biosystems, Foster City, CA, USA) was performed as described by Chang et al. [17]. All of the expression values were normalized to the expression of bll7457 (*hisS*, histidyl t-RNA synthetase), which is a gene constitutively expressed under all conditions used.

### 4.8. Electron Microscopy

Freeze-fractured nodules were fixed in glutaraldehyde (2% final concentration) and examined with a Hitachi S-4700 Scanning Electron Microscope.

## Figures and Tables

**Table 1:** Categories of Expressed and Constitutive Genes. Each identified gene was placed in the corresponding category as defined by Kaneko et al. [22]. The values in parenthesis are the percentage that each category represents of the total number of transcripts in each group (up-regulated, down-regulated, or constitutive). The transcripts included in either the up- (>2-fold) or down-regulated (<2-fold) columns were genes that display the respective values at all six time points.

Category	Upregulated	Downregulated	Constitutive	Total
Amino acid metabolism	9 (1.4%)	32 (3.3%)	17 (1.8%)	58
Cofactors, prosthetic groups, and carriers	13 (2.0%)	20 (2.0%)	12 (1.3%)	45
Cell envelope	4 (0.6%)	28 (2.9%)	14 (1.5%)	46
Cellular processes	15 (2.3%)	66 (6.8%)	60 (6.5%)	141
Central intermediary metabolism	40 (6.1%)	30 (3.1%)	46 (5.0%)	116
DNA replication, recombination, and repair	5 (0.8%)	11 (1.1%)	9 (1.0%)	25
Energy metabolism	19 (3%)	54 (5.5%)	40 (4.3%)	113
Fatty acid, phospholipid, and sterol metabolism	6 (0.9%)	23 (2.4%)	17 (1.8%)	46
Purines, pyrimidines, nucleosides, and nucleotides	2 (0.3%)	15 (1.5%)	11 (1.2%)	28
Regulatory functions	39 (6.0%)	56 (5.7%)	55 (5.9%)	150
Transcription	5 (0.8%)	10 (1.0%)	11 (1.2%)	26
Translation	9 (1.4%)	65 (6.7%)	51 (5.5%)	125
Transport and binding proteins	46 (7.0%)	61 (6.3%)	63 (6.8%)	170
Other functions	72 (11.0%)	81 (8.3%)	78 (8.4%)	231
Hypothetical	369 (56.5%)	424 (43.4%)	443 (47.8%)	1236
Total	653	976	927	2556

**Table 2:** Examples of *B. diazoefficiens* genes expressed during the symbiosis of soybean root nodules following the pattern displayed in Figure 1B.

Function	Rhizobase	Accession	Location
ABC transporter substrate-binding protein	Bll4544	BAC49809	Periplasm
FixK2 protein	Blr2757	CAA06287	Cytoplasmic
C-4 Dicarboxylate transport protein	Bll1718	NP_768358	Cytoplasmic membrane
Iron response regulator	Bll0768	AHY55539	Cytoplasm
Citrate synthase	Blr4839	NP_771479	Cytoplasm
Aspartate aminotransferase	Blr1686	NP_768326	Cytoplasm
Divalent cation resistant protein	Blr4935	NP_771575	Outer Membrane
Alcohol dehydrogenase	Bll4482	NP_771122	Unknown
Taurine Dioxygenase	Bll2125	APO50734	Cytoplasm
Β-Lactamase	Bll2252	AND87779	Cytoplasm
Tripartite tricarboxylate transporter TctB	Bll3050	AND88496	Cytoplasmic membrane
Tripartite tricarboxylate transporter substrate binding protein	Blr3161	AWO90155	Periplasm
3-β hydroxysteroid dehydrogenase	Bll4299	AND89578	Cytoplasm
C4-Dicarboxylate ABC transporter	Blr5025	AND90243	Periplasm
(2Fe-2S)-binding protein	Bll6238	AND91348	Cytoplasm
Hemolysin D	Bll6258	AND91366	Cytoplasmic membrane
Hemolysin secretion protein D	Blr3031	AND88479	Unknown
Dienelactone hydrolase	Bll7509	AWO94311	Unknown
Serine hydrolase	Bll8153	AWO94832	Cytoplasmic membrane
Asp/Glu/hydantoin racemase	Blr3294	AND88720	Unknown
Phospholipase	Blr5550	AWO92367	Unknown
Allophanate hydrolase	Blr3633	AND88989	Cytoplasm
Anhydro-N-acetylmuramic acid kinase	Blr4331	AND89607	Unknown
Dolichol-phosphate mannosyltransferase	Blr4442	NP_771082	Cytoplasmic membrane
ATPase	Blr7361	AND92326	Unknown
2-Hydroxyhepta-2,4-diene-1,7-dioate isomerase	Blr7891	AND92773	Cytoplasm

**Table 3:** Examples of *B. diazoefficiens* genes expressed during the senescence of soybean root nodules following the pattern displayed in Figure 7.

Function	Rhizobase	Accession	Location
Glutamate synthetase I	Blr4835	BAC50100	Cytoplasm
Proline iminopeptidase	Bll4403	BAC49668	Cytoplasm
Pantoate-beta-alanine ligase	Blr6152	BAC50427	Cytoplasm
β-ketoadipyl CoA thiolase	Blr0925	BAC46190	Unknown
Glycerate dehydrogenase	Bll2918	BAC48183	Cytoplasm
Cytochrome C oxidase	Bll3783	BAC49048	Cytoplasmic membrane
Murein endopeptidase	Blr8158	BAC53423	Periplasm
Ribose 5-phosphate isomerase	Blr3755	BAC49020	Cytoplasm
3-Deoxy-manno-octulosonate cytidylyltransferase	Bll1422	BAC46687	Cytoplasm
Sugar ABC transporter permease	Bll2675	BAC47940	Cytoplasmic membrane
Flagellar protein	Bll6868	BAC52133	Extracellular
3-Oxoacyl-(acyl-carrier-protein) synthase II	Bll3809	BAC49074	Unknown
Branched-chain amino acid ABC transporter substrate-binding protein	Bll0979	BAC46244	Periplasm
Cytochrome O ubiquinol oxidase	Blr2715	AND93893	Cytoplasmic membrane
Glycosyl hydrolase	Blr6771	NP_773411	Cytoplasm
Alkylhydroperoxidase	Bl57595	AND92531	Unknown
3-Oxoacyl-ACP reductase	Bll4596	AWO91529	Periplasm
Methyltransferase	Blr2533	AWO89558	Cytoplasm
Patatin-like phospholipase	Bll7964	AWO94740	Extracellular
LysE family translocator	Bll6498	PDT61394	Cytoplasmic membrane
Amidohydrolase	Bll7610	AWO94407	Cytoplasm
Phosphodiesterase	Bll4141	AWO91078	Cytoplasm
Pyridoxamine 5′-phosphate oxidase	Blr4155	AWO91090	Cytoplasm
Peptide ABC transporter permease	Blr0310	BAC45575	Cytoplasmic membrane
TetR-like transcriptional regulator	Bll7298	BAC52563	Unknown
enoyl-CoA hydratase	Bll0116	AWO87220	Cytoplasm

**Table 4:** Examples of *B. diazoefficiens* genes expressed during the senescence of soybean root nodules following the pattern displayed in Figure 8.

Function	Rhizobase	Accession	Location
Citrate-proton symporter	Bll1864	WP_06090909	Cytoplasmic Membrane
Ribose 5-phosphate isomerase	Blr3755	NP_770395	Cytoplasm
Acyltransferase	Blr4337	AND94080	Cytoplasmic Membrane
Thymydilate kinase	Bll4518	NP_771158	Cytoplasm
Homoserine O-succinyltransferase	Bll0244	BAC45509	Cytoplasm
Proline iminopeptidase	Bll4403	NP_771043	Cytoplasm
3-Dehydroquinate dehydratase	Bll4292	NP_770932	Cytoplasm
Epoxide hydrolase	Blr2881	NP_769521	Unknown
ATP-dependent DNA helicase	Bll0242	NP_766882	Cytoplasm
Pyridoxamine 5′-phosphate oxidase	Bll7835	AND92725	Unknown
Beta-ketoadipyl CoA thiolase	Blr0925	NP_767565	Cytoplasm
Branched chain amino acid ABC transporter permease protein	Blr2923	NP_769563	Cytoplasmic Membrane
Sugar ABC transporter permease protein	Bll2675	NP_769315	Cytoplasmic Membrane
3-Deoxy-manno-octulosonate cytidylyltransferase	Bll1422	WP_011084239	Cytoplasm
Exoribonuclease	Blr5112	NP_771752	Cytoplasm
Δ9 acyl-lipid fatty acid desaturase	Bll4594	WP_011087365	Cytoplasmic Membrane
DNA glycosylase	Blr6661	NP_773301	Unknown
Outer membrane channel lipoprotein	Bll4321	NP_770961	Outer Membrane
Cytochrome P450	Bll0557	NP_767197	Unknown
Transcriptional regulatory protein IclR family	Blr3939	NP_770579	Cytoplasm
Purine-binding chemotaxis protein	Blr2193	NP_768833	Cytoplasm
Patatin-like phospholipases	Bll7964	AWO94740	Cytoplasm
Branched-chain amino acid ABC transporter substrate-binding protein	Bll0979	WP_011083799	Periplasm
Phosphoserine aminotransferase	Bll7402	NP_774042	Cytoplasm
Rubrerythrin	Blr7895	KOY06556	Cytoplasmic Membrane

**Table 5:** The frequency of rare codons in each expression pattern and in the transcription and translation functions. Values are frequency per 1000 codons. The lettered superscripts indicate a statistical difference (*p* < 0.5) between similar indicated values. The asterisks (*) indicate that the value of 0 is statistically different from each of the other categories.

Expression Pattern/Function	Rare Codon									
	TTA	TCT	TGT	CTA	ATA	ACT	AGT	AGA	GTA	All
Symbiotic	1.30^ABC^	3.01^ABCD^	2.20^AB^	1.91^AB^	2.66^A^	4.11^ABCD^	3.41^ABC^	3.09^ABC^	2.80^AB^	2.72^ABCDEF^
Post-Symbiotic	0.43^A^	2.31	1.33	2.23^CD^	2.01	3.30^E^	2.45^D^	2.84^DEF^	2.91	2.20^AGHIJ^
Reciprocal Symbiotic	1.07	2.76	1.46	2.18	1.34	3.00^F^	2.30^E^	2.16^G^	2.28	2.06^BK^
Constitutive	0.35^B^	1.06^A^	0.97	0.80^AC^	1.05	1.65^AEF^	1.62^AF^	1.48^AH^	1.89	1.20^CGKLMN^
Transcription	0.49^C^	1.43^B^	0.53^A^	1.20	0.43^A^	2.18^B^	2.49^G^	1.29^BDEI^	1.30^A^	1.26^DHLO^
30S Ribosome Genes	0 *	1.72^C^	0 *	0 *	0 *	2.36^C^	1.07^B^	0.78	0.57^B^	0.72^EIM^
50S Ribosome Genes	0 *	1.82^D^	0.43^B^	0.12^BD^	0 *	2.69^D^	0.29^CDEFG^	0.14^CFGHI^	1.87	0.82^FJNO^

**Table 6:** Frequency of GC, AG, and nucleotide contents within each expression pattern and the transcription and translation functional groups. Values are frequency per 1000 codons. Superscripts indicate a statistical difference (*p* < 0.5) between similar indicated values.

Expression Pattern/Function	%GC	%AG	%A	%T	%G	%C
Symbiotic	63.4^AB^	57.92^ABCDEF^	18.59^AB^	18.19^ABCD^	31.40^AB^	32.02^A^
Post-Symbiotic	63.88^C^	49.64^AG^	18.00^ACD^	18.11^EFGH^	31.64^A^	32.23
Reciprocal Symbiotic	64.13	49.19^BHKL^	17.72^EFG^	18.14^IJK^	31.46	32.66
Constitutive	65.18^ACD^	50.39^CIK^	18.09^BHIJ^	16.77^AEILM^	32.31^BC^	32.87^A^
Transcription	64.94^BE^	51.12^DL^	18.92^EHKL^	16.15^BFJN^	32.16	32.77
30S Ribosome Proteins	63.23^E^	51.93^EJ^	20.97^CDFIK^	15.81^CGL^	30.96^CD^	32.26
50S Ribosome Proteins	63.62^D^	53.08^FGHIJ^	21.30^GJL^	15.00^DHKMN^	31.77^D^	31.92
